# Biased action of the CXCR4-targeting drug plerixafor is essential for its superior hematopoietic stem cell mobilization

**DOI:** 10.1038/s42003-021-02070-9

**Published:** 2021-05-12

**Authors:** Astrid S. Jørgensen, Viktorija Daugvilaite, Katia De Filippo, Christian Berg, Masa Mavri, Tau Benned-Jensen, Goda Juzenaite, Gertrud Hjortø, Sara Rankin, Jon Våbenø, Mette M. Rosenkilde

**Affiliations:** 1 Department of Biomedical Sciences, Faculty of Health and Medical Sciences, The Panum Institute, University of Copenhagen, Copenhagen, Denmark; 2 Department of Medicine, National Heart and Lung Institute (NHLI), Imperial College, London, United Kingdom; 3 Unit for Infectious Diseases, Department of Medicine, Herlev-Gentofte Hospital, University of Copenhagen, Herlev, Denmark; 4 Institute of Preclinical Sciences, Veterinary Faculty, University of Ljubljana, Ljubljana, Slovenia; 5 Helgeland Hospital Trust, Sandnessjøen, Norway; 6 Lundbeck A/S, Copenhagen, Denmark

**Keywords:** Molecular medicine, Receptor pharmacology, Haematopoietic stem cells

## Abstract

Following the FDA-approval of the hematopoietic stem cell (HSC) mobilizer plerixafor, orally available and potent CXCR4 antagonists were pursued. One such proposition was AMD11070, which was orally active and had superior antagonism in vitro; however, it did not appear as effective for HSC mobilization in vivo. Here we show that while AMD11070 acts as a full antagonist, plerixafor acts biased by stimulating β-arrestin recruitment while fully antagonizing G protein. Consequently, while AMD11070 prevents the constitutive receptor internalization, plerixafor allows it and thereby decreases receptor expression. These findings are confirmed by the successful transfer of both ligands’ binding sites and action to the related CXCR3 receptor. In vivo, plerixafor exhibits superior HSC mobilization associated with a dramatic reversal of the CXCL12 gradient across the bone marrow endothelium, which is not seen for AMD11070. We propose that the biased action of plerixafor is central for its superior therapeutic effect in HSC mobilization.

## Abstract (web summary)

Jørgensen et al. investigate the effects of the CXCR4 targeting agents, AMD3100 (Plerixafor) and AMD11070, and show that AMD3100, unlike AMD11070, is biased with intrinsic β-arrestin recruitment agonism and full G protein antagonism. They finally propose that this biased action of AMD3100 is central for its superior therapeutic effect on mobilizing stem cells.

## Introduction

G protein-coupled receptors (GPCRs) are the largest family of membrane proteins in the human body. Due to their important roles in physiology and pathophysiology, GPCRs are highly desirable drug targets and are currently targeted by approximately one-third of marketed drugs^1^. Agonist binding leads to rearrangement of the intracellular domains, which enables coupling to signal transducers. In addition to the canonical signaling pathway mediated by heterotrimeric G proteins, activated GPCRs are often phosphorylated by GPCR kinases (GRKs), which initiates arrestin recruitment, receptor desensitization, internalization, recycling/degradation, and/or intracellular signaling^2^. G proteins, GRKs, and arrestins all engage the intracellular cavity of the GPCR, and subtle differences in the conformational receptor ensemble stabilized by the agonist determine the preferred interaction partner(s)^3^. Thus, the functional outcome of ligand binding ultimately depends on the structural characteristics of the ligand:receptor complex. When a ligand selectively activates or inhibits one of several signaling pathways, the ligand is said to be biased. A better understanding of ligand bias will allow the design of drugs that target only a subset of a receptor’s functions, resulting in more precise therapeutic effects and/or fewer side-effects. Thus, tailoring of GPCR-ligands that selectively affect β-arrestin- or G protein signaling events is pursued within many different physiological systems^4^.

CXC chemokine receptor 4 (CXCR4) belongs to the chemokine receptor subfamily of class A GPCRs^5^. It was initially cloned based on its role as HIV cell-entry co-factor, a property it shares with CC chemokine receptor 5 (CCR5)^6^. CXCR4 is widely expressed in human tissue and involved in diverse biological processes such as angiogenesis^7^, embryonic development^8^, homing regulation of HSCs^9,10^, metastasis^11–14^, and immune cell chemotaxis towards its endogenous ligand CXC chemokine ligand 12 (CXCL12).

**Fig. 1:**
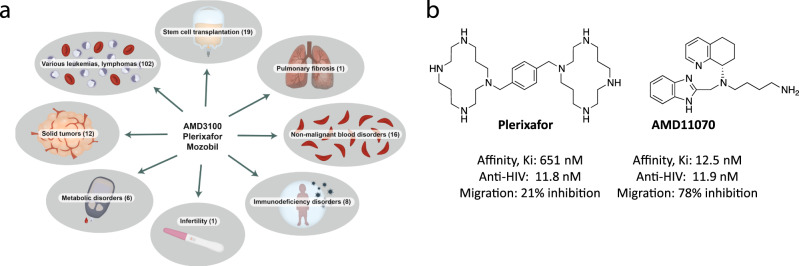
Clinical trials involving plerixafor, and comparison of plerixafor vs AMD11070. **a** Overview of the types of clinical trials where plerixafor have been tested. The numbers in brackets show the number of trials within the different therapeutic areas. In scientific literature, plerixafor is also known as AMD3100. The drug is used under the trade name Mozobil. This image was created by Anna Sofie Husted using Adobe illustrator and photoshop under the licence of University of Copenhagen. **b** Chemical structures of plerixafor and AMD11070. The values below show their binding affinity for CXCR4 measured as their ability to inhibit CXCL12 binding in competitive binding^22^, their ability to inhibit viral replication in MT-4 cells infected with the HIV-1 strain^21^, and their ability to block migration of the A375 melanoma cell line towards CXCL12 (10 nM)^23^.

The bicyclam CXCR4 antagonist plerixafor (known in the literature as AMD3100) was originally developed to inhibit cell entry of HIV X4-strains via CXCR4; however, initial in vivo preclinical tests revealed a massive leukocytosis following plerixafor administration^15^. This observation resulted in the launch of plerixafor (trade name Mozobil) as a first-in-class HSC mobilizing compound for the autologous transplantation of bone marrow (BM) cells in patients with Non-Hodgkin’s lymphoma and multiple myeloma^15^. Up to now, plerixafor has been tested in 165 clinical trials (Supplementary Data 1) mainly with HSC recruitment for BM transplants as the primary clinical endpoint, but also for other indications such as Warts, Hypogammaglobulinemia, Infections, and Myelokathexis (WHIM) syndrome, solid tumors, metabolic, cardiovascular, and pulmonary disorders^16^ (Fig. 1a). Following the launch of plerixafor, which is administered subcutaneously, several other CXCR4 antagonists were developed and entered clinical trials^17^. One of these was the potent and orally available small-molecule AMD11070 (Fig. 1b), which was initially tested in the clinic as anti-HIV treatment^18–20^. More recently, clinical trials of AMD11070 (also known as X4P-001 and mavorixafor) have been conducted for certain types of cancers, e.g., clear cell renal cell carcinoma and melanoma (ClinicalTrials.gov Identifiers: NCT02667886 and NCT02823405, respectively), as well as for WHIM syndrome, where it currently is in Phase 3 (ClinicalTrials.gov Identifier: NCT03995108).

Plerixafor and AMD11070 both block CXCL12 activity, but with different efficacies. While they are equipotent in their ability to inhibit HIV viral replication^21^, AMD11070 displays a much higher CXCR4 affinity than plerixafor when measured in CXCL12 competitive binding assays^22^. Similarly, AMD11070 is much more efficient in blocking CXCL12-mediated chemotaxis^23^.

A gradient of CXCL12 across the BM endothelium regulates the homing of HSC, and the higher chemokine levels in the BM retain the CXCR4 positive HSC within this compartment. This gradient is regulated by endothelial expressed CXCR4, which facilitates active transport of CXCL12 across the BM endothelium. It has been hypothesized that blocking of CXCR4 by plerixafor, and other CXCR4 antagonists, prevents the CXCR4-mediated transport of CXCL12. This reduces the CXCL12 levels within the BM and reverses the CXCL12 gradient across the BM endothelium, which ultimately leads to HSC release from the BM^10,24–26^. While no parallel study of their white blood cell mobilizing properties has been reported, individual studies showed that blood levels of these cells increased 2.5 fold in response to plerixafor^15,27^, while a lower increase, 1.4 fold, was reported in response to AMD11070^21^. The fact that plerixafor displays a lower CXCR4 affinity than AMD11070^22^, and is less efficient in inhibiting CXCL12-induced migration^23^ suggests that the stem cell mobilization cannot be solely ascribed to disruption of the CXCR4/CXCL12 interaction, and points towards an additional, yet unidentified mechanism of action for plerixafor.

On this background, we investigate the constitutive and ligand-dependent CXCR4 internalization pattern as well as CXCR4-mediated β-arrestin recruitment and show that plerixafor, in contrast to AMD11070, is biased with intrinsic β-arrestin recruitment agonism and full G protein antagonism. These findings are confirmed by the successful transfer of both ligands’ binding sites to CXCR3, where the same biased action of plerixafor, and unbiased action of AMD11070, are observed. Finally, we compare the in vivo cell mobilization properties of the two compounds in the first head-to-head study for this property and show that despite being a less potent CXCR4 inhibitor, plerixafor is indeed superior to AMD11070 in its ability to mobilize HSC. Biased drugs are normally pursued to relieve adverse events; however, we propose that the biased action of plerixafor is central for its superior therapeutic effect on mobilizing stem cells.

## Results and discussion

### Constitutive and ligand-mediated internalization of CXCR4 by CXCL12 and plerixafor

Given the function of CXCR4 and CCR5 as HIV cell entry co-factors, multiple studies were carried out in the nineties to describe the internalization patterns of these two receptors^28–31^. From these, it was shown that CXCR4 is rapidly internalized upon CXCL12 stimulation^28,32–34^. Interestingly, CXCR4 also internalized in the presence of phorbol-ester, suggesting that protein kinase C (PKC) phosphorylation is involved in the internalization process^28^. Following PKC-mediated internalization, the receptor rapidly recycles back to the plasma membrane^28^. In contrast, CXCL12 only poorly induces recycling^35^ as explained by ubiquitination and subsequent sorting to lysosomes and degradation^2,36^—a difference ascribed to variations in intracellular phosphorylation patterns of CXCR4.

**Fig. 2:**
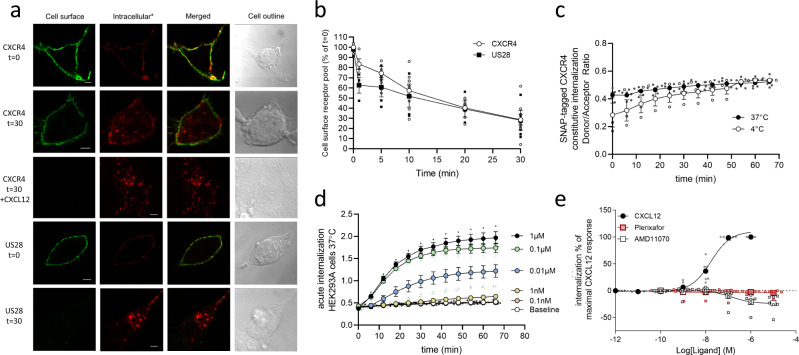
Internalization of CXCR4 is differentially affected by CXCL12, plerixafor, and AMD11070. **a** Antibody feeding experiment with FLAG-tagged CXCR4 and US28 in HEK293 cells. The cells were either immediately fixed (*t* = 0) or incubated at 37 °C for 30 min to induce internalization (*t* = 30) and then fixed. CXCL12 was added at 1 μM. Cell surface receptors are labeled with Alexa Fluor 488-conjugated secondary antibody (green, left column), while internalized receptors were labeled with Alexa Flour 568-conjugated secondary antibody (red, second column from left). Nomarski images are included to illustrate the cell outline (right column). Scale bar, 5 μm. Images show representative cells from three independent experiments. **b** Acute time-course internalization ELISA. HEK293 cells were transiently transfected with FLAG-tagged CXCR4 (white circle) or US28 (black square). For each receptor, the value at a given time point is corrected for background, normalized to the value at *t* = 0, and presented in percent with mean ± SEM shown. The experiment was performed at least four times in quadruples. **c** SNAP-tag CXCR4 constitutive internalization. Internalization was determined after preincubation with Tag-lite SNAP-Lumi4-Tb (donor) for 1 h at 4 °C (white circle) or 37 °C (black circle) and shown with mean ± SEM from at least four independent experiments performed in triplicates. **d** SNAP-tag CXCR4 agonist CXCL12 induced internalization in HEK293A cells. Cells were stimulated with increasing concentration of CXCL12; 0 nM (white circle), 0.1 nM (orange circle), 1 nM (yellow circle), 0.01 µM (blue circle), 0.1 µM (green circle) or 1 µM (black circle). Internalization was determined upon CXCL12 addition after 1-hour preincubation with Tag-lite SNAP-lumi4-Tb at 37 °C. Data are shown with mean ± SEM of triplicates from at least three independent experiments. **e** SNAP-tagged CXCR4 receptor internalization after stimulation with CXCL12 (black circle), plerixafor (red square), and AMD11070 (white square). The data were normalized to maximal internalization levels by CXCL12 for CXCR4 and presented with mean ± SEM of triplicates from at least six independent experiments.

To determine the impact of the two antagonists on CXCR4 internalization, we first adapted an antibody-feeding method using a FLAG-tagged CXCR4 to detect the degree of constitutive internalization (Fig. 2a). The human cytomegalovirus-encoded chemokine receptor US28, which has a fast and ligand-independent internalization^37^, was included as a control. At time 0, both receptors were detected on the cell membrane (Fig. 2a). After 30 min incubation at 37 °C, US28 was no longer present at the cell membrane but could be detected upon cell permeabilization as red puncta inside the cell. CXCR4 also internalized constitutively, although still present at the cell membrane after 30 min. Upon addition of 1 µM CXCL12, CXCR4 internalized to form a high number of puncta within the cell body, with no detection of the receptor on the cell membrane after 30 min. An ELISA-based quantification of the cell surface-expressed receptors over time showed that both CXCR4 and US28 internalized gradually with only around 30% of both receptors still residing on the cell surface after 30 min (Fig. 2b). To further determine the time-course of internalization, we established a real-time internalization assay using an N-terminally SNAP-tagged CXCR4. This allowed us to determine constitutive internalization by preincubation of the cells at 4 °C and agonist-induced internalization by the addition of CXCL12. Consistent with the antibody-feeding experiments, CXCR4 internalized constitutively with a halftime *T*_1/2_ of 22.1 min (Fig. 2c), while 1 µM CXCL12 induced faster internalization with a *T*_1/2_ of 14.1 min (Fig. 2d). The dose-dependent internalization upon CXCL12 stimulation revealed a potency (pEC_50_) of 7.7 ± 0.07 (Fig. 2e), consistent with previous reports^33,34^. In contrast, AMD11070 effectively prevented CXCR4 internalization and thereby resulted in an increased receptor expression with a pEC_50_ of 6.8 ± 0.4 (Fig. 2e). This is a classical behavior for class A GPCR antagonists and inverse agonists, that, by constraining the receptor in its inactive conformations, prevent constitutive receptor internalization^38–40^ and thereby increase receptor surface expression. Importantly, this was not the case for plerixafor, as this compound did not alter the constitutive internalization of CXCR4, but simply allowed it to take place (Fig. 2e).

### Plerixafor induces arrestin recruitment via CXCR4

**Fig. 3:**
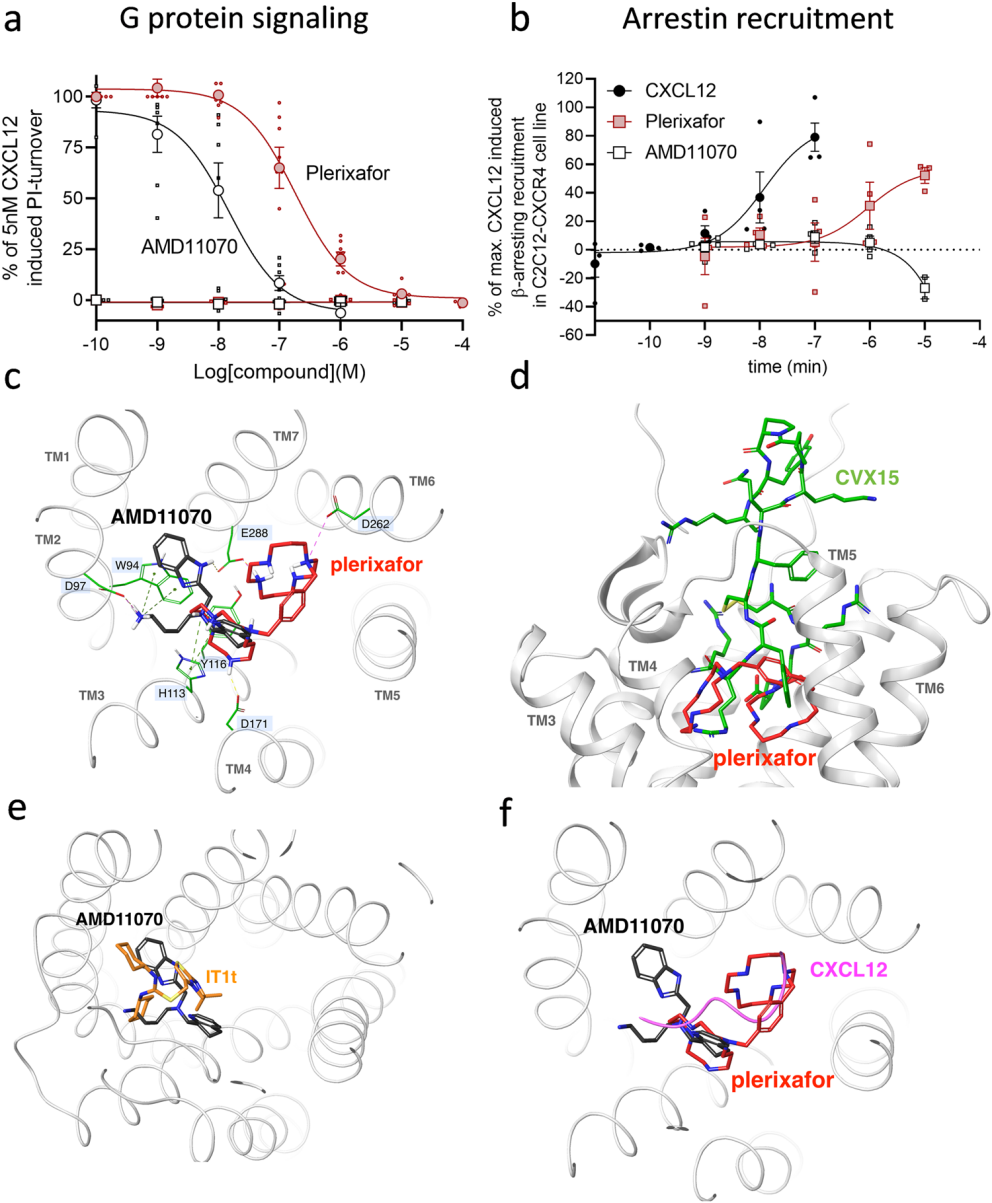
Plerixafor and AMD11070 show distinct pharmacological profiles at CXCR4 and display different CXR4 binding modes. **a** Agonism and antagonism of CXCR4 induced G protein activity. Measured by inositol triphosphate (IP3) accumulation in HEK293 cells transiently transfected with CXCR4 and the chimeric G protein Gqi4myr in the absence (square) or presence (circle) of 5 nM CXCL12 (corresponding to 80% activity). Cells were stimulated with increasing concentration of plerixafor (red) or AMD11070 (white). The data were normalized to signaling levels in response to 5 nM CXCL12 and presented with %mean ± SEM of duplicates from at least three independent experiments. **b** β-arrestin recruitment to CXCR4 by plerixafor (red square) and AMD11070 (white square). CXCL12 (black circle) was used as a control. β-arrestin2 recruitment was measured in C2C12 cells stably expressing Prolink (PK)-tagged CXCR4 and Enzyme Acceptor (EA)-tagged β-arrestin2. The data were normalized to the maximal recruitment levels by CXCL12 and presented with %mean ± SEM of duplicates from four independent experiments. **c** Overlay of the proposed binding modes for plerixafor (red) and AMD11070 (black) in the full-length CXCR4 model (extracellular view). CXCR4 transmembrane helices are shown and annotated in gray. Key binding residues are annotated on a light blue background and shown as sticks in green. Interactions: ionic = dotted magenta; H-bonds = dotted yellow; cation-pi = dotted green. **d** Overlay of the proposed binding mode for plerixafor (red) and the experimental binding mode for the peptide CXCR4 antagonist CVX15 (green; PDB 3OE0) (side view). **e** Overlay of the proposed binding mode for AMD11070 (black) and the experimental binding mode for the small-molecule CXCR4 antagonist IT1t (orange; PDB 3ODU) (viewed as in Fig. 3c). **f** The suggested path of the distal CXCL12 N-terminus (magenta) overlaid on plerixafor (red) and AMD11070 (black) (viewed as in Fig. 3c).

Arrestin recruitment is important for the function of CXCR4^41–44^, and the ability to recruit arrestin is linked to the internalization pattern of CXCR4^28,33,34^. Driven by the different impacts of plerixafor and AMD11070 on CXCR4 internalization, we moved on to determine their role in arrestin recruitment. As previously established^22^, both ligands acted as full antagonists of CXCL12-induced G protein signaling with potencies (pIC_50_) of 6.7 ± 0.09 and 7.8 ± 0.14, respectively (Fig. 3a), again demonstrating the higher potency of AMD11070. As expected from previous data^45^ CXCR4 recruited β-arrestin upon CXCL12 stimulation with high potency (pEC_50_ of 7.7 ± 0.16). However, to our surprise, plerixafor also elicited agonistic properties in β-arrestin recruitment with a potency similar to its inhibition of CXCL12 signaling (pEC_50_ of 6.8 ± 0.75) and an efficacy reaching 30% of 100 nM CXCL12 (Fig. 3b), whereas this was not the case for AMD11070.

Thus, our in vitro data show that plerixafor and AMD11070 both completely abrogate CXCL12-induced Gα_i_ signaling via CXCR4 (Fig. 3a), but in contrast to AMD11070, plerixafor on its own stimulates β-arrestin recruitment (Fig. 3b) and does not block the constitutive internalization of CXCR4, as observed for AMD11070 (Fig. 2e). The internalization is predicted to mediate an even more efficient shutdown of CXCL12-mediated signaling via the removal of CXCR4 from the cell surface. A recent study by Hitchinson et al. supports the importance of an active β-arrestin recruitment arm of CXCR4 for the continued effect of a drug, and thus counteraction of drug tolerance^46^. The authors compared the biased CXCR4 antagonism of plerixafor and X4-2-6, which is a peptide derived from CXCR4 transmembrane (TM) helix 2 and extracellular loop 1. The study demonstrated that X4-2-6 displaced only part of the CXCL12:CXCR4 interaction through the creation of a ternary complex between the peptide, CXCL12, and CXCR4 that disturbs Gα_i_ signaling, but not β-arrestin recruitment. In contrast, plerixafor was shown to displace CXCL12 completely, as also shown previously^46,47^.

### Different binding modes of plerixafor and AMD11070 in CXCR4

The different pharmacological profiles of plerixafor and AMD11070 imply that these two compounds stabilize different conformations of CXCR4, which in turn must relate to differences in binding mode. Neither compound has been co-crystallized with CXCR4, but residues of importance for the binding and function of these (and structurally related) compounds have been identified through extensive site-directed mutagenesis studies, and all of them are located within the TM bundle. The polycationic bicyclam plerixafor has been shown to depend strongly on the acidic residues Asp171^4.60^, Asp262^6.58^, and Glu288^7.39^ ^48–51^, while initial studies of AMD11070 showed strongest dependency on Trp94^2.60^, Asp97^2.63^, Asp171^4.60^, and Glu288^7.39^ ^50^; more recently, the importance of Glu288^7.39^ for AMD11070 antagonism has been confirmed, while the role of Asp171^4.60^ was questioned^22^ (superscripts indicate Ballesteros–Weinstein numbering for conserved GPCR residues^52^). While the experimental data for AMD11070 are somewhat conflicting, they still point to a strong dependency on Glu288^7.39^ and the involvement of residues in the top of TM2 (Trp94^2.60^ and Asp97^2.63^).

To identify plausible binding modes for plerixafor and AMD11070, both compounds were docked to the recently published full-length model of CXCR4^53^ using the induced fit protocol developed by Schrödinger^54^. Since the literature suggests that Glu288^7.39^ is a common interaction site for plerixafor and AMD11070, this residue was used as a constraint for both compounds. For the large and flexible plerixafor, Asp171^4.60^ was used as an additional constraint. Among the generated poses for plerixafor, one was identified where the ligand was curled up in the major binding pocket, forming extensive ionic and H-bond interactions with Asp171^4.60^, Asp262^6.58^, and Glu288^7.39^ (Fig. 3c), which is consistent with experimental data^47,49,51^. Additional cation–π interactions with aromatic residues in TM3 (His113^3.29^, and Tyr116^3.32^) were also seen. This binding mode for plerixafor shares several contact residues with the bottom part of the peptide antagonist CVX15 (Fig. 3d), which has been co-crystallized with CXCR4^55^. The majority of the poses for AMD11070 were confined to the minor binding pocket, and in the top-scoring pose, the protonated primary and tertiary amines form ionic interactions with Asp97^2.63^ and Glu288^7.39^, respectively, as well as cation–π interactions with flanking aromatic residues (Trp94^2.60^, His113^3.29^, and Tyr116^3.32^) (Fig. 3c). Glu288^7.39^ is also involved in a charge-assisted H-bond with the benzimidazole ring. In this binding mode, AMD11070 is located in the same region as the small-molecule antagonist IT1t (Fig. 3e)^55^.

Using the X-ray structure of bovine rhodopsin as a homology template for CXCR4, Fricker et al. previously proposed three alternative binding modes for both plerixafor and AMD11070^50^. The same group subsequently showed that AMD11070 behaves as an allosteric inhibitor with respect to CXCL12, and suggested that this behavior could reflect a combination of multiple (orthosteric and allosteric) binding modes for AMD11070^20^. While the exact binding mode for CXCL12 itself has not yet been elucidated, we consider the CXCL12:CXCR4 complex used in the present study^53^ to be the most mature model to date. Our proposed binding modes for plerixafor and AMD11070 (Fig. 3c) are both in steric conflict with the suggested position of the CXCL12 N-terminus in the so-called chemokine recognition site 2 (CRS2) within the TM bundle (Fig. 3f)^53^, which defines both compounds as orthosteric antagonists. Thus, we believe that the apparent allosteric behavior of AMD11070 is an example of “orthosteric allostery”, which is commonly observed for small-molecule chemokine antagonists that bind to the large orthosteric CRS2^56^.

### Transferring the binding sites of plerixafor and AMD11070 from CXCR4 to CXCR3

**Fig. 4:**
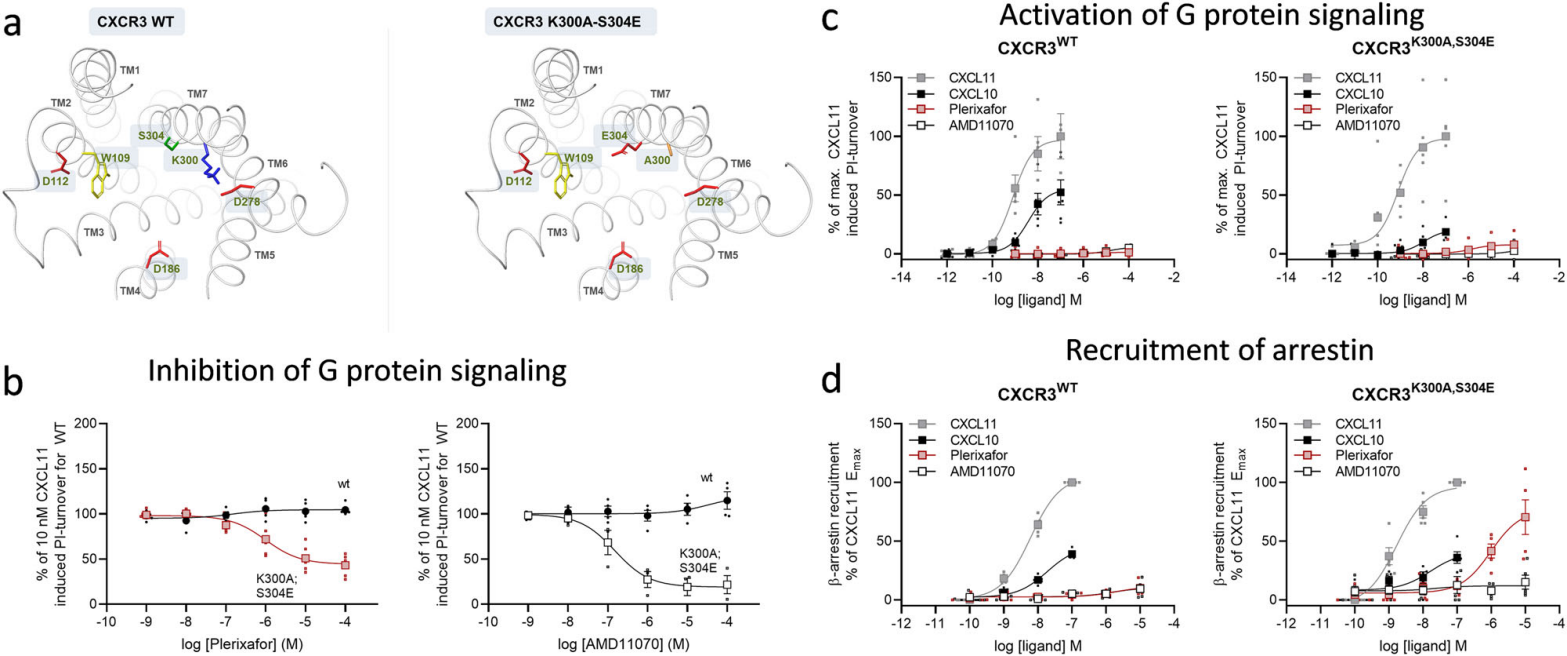
Transfer of plerixafor to CXCR3 (also) reveals partial agonism. **a** Binding pocket of WT CXCR3 (left) and K300A-S304E mutant (right) shown from the extracellular side. Residues involved in plerixafor and AMD11070 binding in CXCR4 (Fig. 3) are shown as sticks. K300 and S304 in WT are mutated to A300 and E304, respectively. The wild-type CXCR3 model was retrieved from the GPCR-HGmod database^76^. Residues are colored according to side chains; red = acidic residue, blue = basic residue, yellow = aromatic residue, green = polar residue. **b** Antagonistic effect of plerixafor and AMD11070 on CXCL11-induced CXCR3 G protein activity. Measured by inositol triphosphate (IP3) accumulation in HEK293 cells transiently transfected with WT CXCR3 or CXCR3[K300A-S304E] and the chimeric G protein Gqi4myr and stimulated with 10 nM CXCL11. Data of plerixafor previously published in ref. ^48^. Data are shown with %mean ± SEM of duplicates from 3 independent experiments, CXCR3 wt (black circle) and CXCR3 [K300A-S304E] stimulated with plerixafor (red square) or AMD11070 (white square). **c** Agonistic effect of plerixafor and AMD11070 on CXCR3 G protein activity compared to CXCL10 and CXCL11. Measured by inositol triphosphate (IP3) accumulation in HEK293 cells transiently transfected with CXCR3 WT or CXCR3[K300A-S304E] and the chimeric G protein Gqi4myr. The data were normalized to the maximal recruitment levels by CXCL11 on the two receptors and presented with %mean ± SEM of duplicates from at least three indepependent experiments. **d** Agonistic effect of plerixafor and AM11070 in β-arrestin2 recruitment upon CXCR3 activation compared to CXCL10 and CXCL11. Measured by the BRET-based arrestin recruitment assay in CHO cells transiently transfected with WT CXCR3 or CXCR3[K300A-S304E], Rluc8-Arrestin3, and mem-linker-citrine-SH3 constructs. The data were normalized to the maximal recruitment levels by CXCL11 on the two receptors and presented with %mean ± SEM of duplicates from five independent experiments. In **c** and **d** data are presented as CXCL11 (gray square), CXCL10 (black square), plerixafor (red square), and AMD11070 (white square).

The CXCR4 binding site of plerixafor has previously been successfully introduced in CXCR3 by the double mutation K300A-S304E, making the compound an efficient antagonist of CXCL10 and −11 induced G protein activity^48^. However, its effect on arrestin recruitment has not been assessed in this modified CXCR3 receptor, nor has AMD11070 antagonism of CXCR3 been assessed. Sequence alignment of the two receptors shows that most of the suggested contact residues for plerixafor and AMD11070 in CXCR4 (Fig. 3c) are conserved in CXCR3, including three out of the four acidic residues identified as anchor points for plerixafor and/or AMD11070: Asp112^2.63^, Asp186^4.60^, and Asp278^6.58^ (Fig. 4a). However, instead of a glutamate residue in position 7.39, which is otherwise highly conserved in the chemokine receptor family^57^, CXCR3 contains Ser304^7.39^. We constructed the previously published CXCR3 mutant K300A-S304E^48^ to introduce the missing negatively charged interaction site, Glu^7.39^, and to remove a potential steric and/or electrostatic repulsion from a neighboring positively charged side chain (Lys300^7.35^) within the binding site (Fig. 4a). These modifications enabled both plerixafor (shown previously^48^) and AMD11070 to antagonize G protein activation upon stimulation of the double CXCR3 mutant with CXCL11 (Fig. 4b) and CXCL10 (Supplementary Fig. 1)—similar to the antagonistic effect on CXCL12-induced CXCR4 activity (Fig. 3a). Analogous to their antagonistic potency on CXCR4, AMD11070 was a more potent inhibitor of CXCL11-induced activity than plerixafor in the CXCR3 mutant, with pIC_50_ values (mean ± SEM) of 6.8 ± 0.21 and 6.0 ± 0.21, respectively. Neither plerixafor nor AMD11070 induced any G protein signaling on their own (Fig. 4c).

### Similar bias of plerixafor and full antagonism of AMD11070 in the engineered CXCR3

We next assessed the recruitment of arrestin for the modified CXCR3 construct. The capacity of CXCR3 to recruit β-arrestin in response to both CXCL10 and CXCL11 was maintained in the double mutant (Fig. 4d). AMD11070 was not able to induce arrestin recruitment in either the WT or mutant receptor; however, while WT CXCR3 did not respond to plerixafor, the S304E-K300A CXCR3 mutant recruited β-arrestin2 in response to plerixafor to an even higher level than CXCL10 (Fig. 4d). Thus, plerixafor and AMD11070 displayed similar pharmacological profiles at both WT CXCR4 and the double CXCR3 mutant, where both compounds antagonized G-protein signaling, and plerixafor—in contrast to AMD11070—recruited β-arrestin.

While structural studies have established the outward movement of the intracellular end of TM6, and to a lesser extent TM5, as a central event in G protein-signaling^58^, experimental structures of GPCR:arrestin complexes have only emerged recently (visual arrestin:rhodopsin^59,60^, *β*-arrestin in complex with M2 muscarinic receptor (M2R)^61^ and the neurotensin receptor 1 (NTSR1)^62^). While these structures have provided new insights into the details of the GPCR:arrestin interface, they did not reveal a novel arrangement of the intracellular TM cavity that explains the arrestin/G protein selectivity. However, arrestin recruitment requires phosphorylation of the intracellular GPCR domains, and therefore also depends on the engagement of GRKs with the intracellular TM cavity. Consequently, the biased action observed for plerixafor could be a result of increased GRK engagement. While the molecular details of the GPCR:GRK complex remain unknown, the literature on GPCR transduction points to a central role of the TM helices that form the major binding pocket (TMs 3-7).

The successful transfer of the biased action of plerixafor in CXCR4 to the engineered CXCR3 not only verifies the suggested binding modes for plerixafor and AMD11070 in CXCR4 but also points to a more general mechanism for the bias. While AMD11070 mainly engages TM helices in the minor pocket, we propose that binding of plerixafor to the major binding pocket of CXCR4 induces subtle rearrangements in the intracellular parts of these TM helices, which enables GRK engagement and subsequent *β*-arrestin recruitment.

### In vivo effect of plerixafor and AMD11070 on hematopoietic stem cell and neutrophil mobilization

Having established that plerixafor indeed displays a different pharmacological profile than the pure antagonistic AMD11070, we next looked into their ability to affect the CXCL12 gradient across the BM endothelium, which is directly related to the ability to mobilize hematopoietic progenitor cells (HPC) and neutrophils from the BM.

**Fig. 5:**
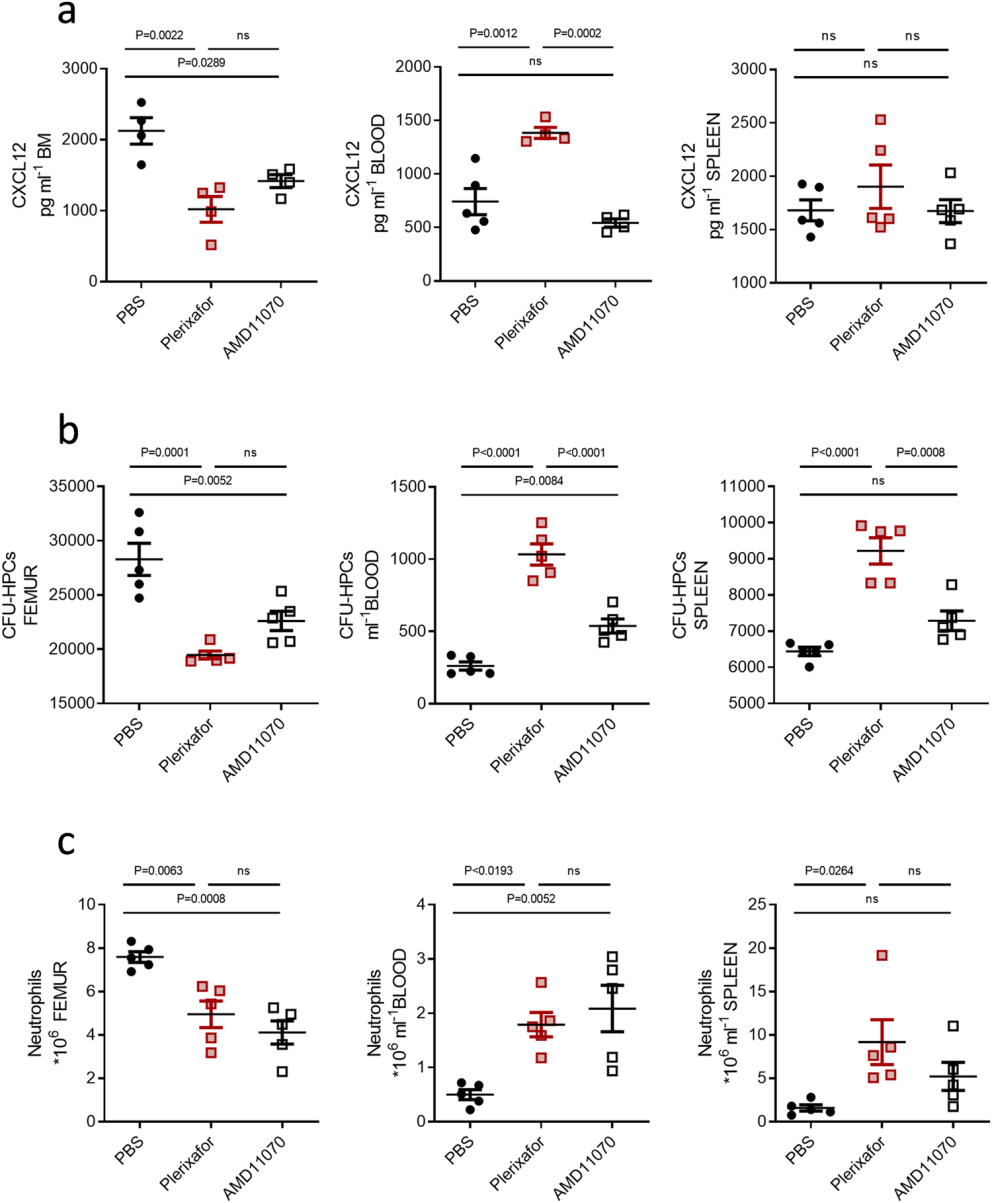
In vivo effect of plerixafor and AMD11070 on HPC and neutrophil mobilization from the BM and redistribution. **a** CXCL12 ELISA on BM and spleen supernatant and blood serum. Values display the concentration of CXCL12 as pg ml^−1^(*n* = 4–5). **b** Counting of CFU-HPCs (colony forming units, hematopoietic progenitor cells) after 12 days of culture following cell harvesting from bone marrow (BM), blood, and spleen, values displayed as cells ml^−1^ (*n* = 5). Photos of cultures can be found in Supplementary Fig. 3. **c** FACS analysis of the total number of neutrophils in the femur, blood, and spleen 1 h after i.p. injection of PBS, plerixafor, or AMD11070 (*n* = 5). The gating strategy for flow cytometry can be found in Supplementary Fig. 3. Color codes throughout the figure are PBS (black circles), Plerixafor (red squares), AMD11070 (white squares). Mean values and error bars representing SEM are shown. Statistical significances were analyzed by one-way ANOVA, exact *P*-values are shown for each comparison, ns = *P* ≥ 0.05.

Consistent with previous studies, plerixafor treatment appeared to reverse the CXCL12 gradient across the BM by greatly decreasing the chemokine levels in the BM, and concomitantly increasing the levels within the blood (Fig. 5a). AMD11070 on the other hand did not reverse the gradient. Treatment with this compound significantly reduced the CXCL12 chemokine levels in the BM but did not lead to an increase in the plasma levels of the chemokine. This implies that both plerixafor and AMD11070, through blocking of CXCR4, can inhibit the active transport of CXCL12 across the BM endothelium, thereby reducing the chemokine levels within the BM. However, as AMD11070 is a more potent CXCR4 inhibitor than plerixafor, the greater impact of plerixafor on the CXCL12 levels might be the result of its biased action. By inducing arrestin recruitment and facilitating endocytosis of CXCR4, plerixafor not only blocks CXCR4 from CXCL12 binding but also induces intracellular scavenging of the receptor, resulting in lower receptor expression and thus a much lower ability to actively transfer CXCL12 into the BM. In fact, surface expression analysis showed that plerixafor led to a decreased CXCR4 surface expression, whereas AMD11070 did not (Supplementary Fig. 2).

As plerixafor and AM11070 have never been directly compared for their ability to mobilize hematopoietic stem and progenitor cells from the BM, we next looked into how these compounds affected the number of HPC and neutrophils in the BM, blood, and spleen (Fig. 5b, c). Plerixafor lowered the number of stem cells in the BM, as the number of colony-forming units from HPCs (CFU-HPC) was lower for BM-derived cells in mice treated with plerixafor compared to PBS (Fig. 5b). This was also evident when visually inspecting the colonies formed by the flushed-out cells (Supplementary Fig. 3). While plerixafor lowered the CFU-HPC number for the BM, the numbers increased for both the blood- and spleen-derived cells, indicating that the mobilized stem cells gather in these compartments. Treatment with AMD11070 also led to a decrease of CFU-HPC in the BM, and an increase of HPC in the blood, but to a much lesser extent than plerixafor. While plerixafor led to an almost 4 fold increase in the CFU-HPC count in the blood (mean CFU-HPC ml^−1^ ± SEM of 261 ± 29 for PBS compared to 1033 ± 73 for plerixafor), AMD11070 only doubled the number (538 ± 49). Moreover, AMD11070 did not increase the CFU-HPC count for cells derived from the spleen.

The mobilization of stem cells from the BM upon treatment with either compound also corresponded to a change in neutrophil numbers in the distinct compartments. Although, here AMD11070 and plerixafor were equipotent with respect to the reduction in the neutrophil numbers in the BM and the increase of the numbers in the blood (Fig. 5c). Treatment with both compounds increased the numbers of neutrophils in the spleen, although the increase was only significant for plerixafor. The increased splenic neutrophil numbers may reflect pooling of cells in the spleen as a way of regulating circulating neutrophil numbers^27^. Taken together these data suggest that plerixafor is more potent than AMD11070 with respect to mobilization of HPCs whereas they might be equipotent with respect to neutrophil mobilization.

Mature neutrophils in the BM reserve express very low levels of CXCR4^63^ and are thus very sensitive to small changes in CXCL12 levels. Therefore, even though AMD11070 induces smaller changes in CXCL12 levels it might still be sufficient to cause neutrophil mobilization from the BM, explaining why the two compounds are equipotent in neutrophil mobilization. In contrast, HPCs express relatively high levels of CXCR4 and therefore may require a more dramatic change in CXCL12 to affect their mobilization. Thus, AMD11070 causes a reduction in CXCL12 in the BM that is sufficient to cause a modest mobilization of HPC to the blood. However, the complete reversal of the chemokine gradient in response to plerixafor leads to a robust stem cell mobilization with the number of circulating HPCs significantly exceeding that observed for AMD11070, and with an excess of HPC mobilizing to the spleen.

## Conclusion

The central role of the CXCL12:CXCR4 axis in the regulation of HPC numbers in the BM is confirmed by the strong HPC mobilizing properties of the CXCR4-targeting antagonist plerixafor that efficiently blocks CXCL12-mediated CXCR4 signaling^8,9^. It is thought that plerixafor acts as a mobilizer by reversing the gradient of CXCL12 formed across the BM and blood through its blockage of a CXCR4-mediated CXCL12 transport across the endothelium^10,24,64,65^.

Since plerixafor inhibits both CXCL12-induced G protein signaling and CXCL12-induced *β*-arrestin/internalization of CXCR4, it has been classified in the literature as an unbiased CXCR4 antagonist^46,66^. We have previously shown that plerixafor itself is devoid of agonistic properties in G protein activation^22^; however, to our knowledge, the present study is the first to report the effects of plerixafor alone on CXCR4 arrestin recruitment and internalization.

Concomitant with the ability of plerixafor to induce arrestin recruitment this compound also allows the constitutive internalization of CXCR4—and might itself lead to further receptor endocytosis. Thus, plerixafor leads to scavenging of the CXCR4 receptor from the cell surface. The BM endothelium expresses CXCR4 and has previously been shown to support the transcytosis of CXCL12 from the plasma into the BM^25^. We suggest that by both antagonizing CXCR4 and reducing CXCR4 expression this process is effectively inhibited, reversing the CXCL12 chemokine gradient across the BM. In contrast, AMD11070 as a pure antagonist blocks the constitutive receptor internalization and may instead facilitate an increased receptor expression by supporting receptor recycling to the surface, allowing the chemokine transport to occur to some degree.

On a molecular level, the pharmacological differences between the two compounds relate to differences in binding modes. While site-directed mutagenesis studies have shown that plerixafor and AMD11070 share dependency on Glu288^7.39^ in the middle of the TM binding pocket, plerixafor shows selective dependency on Asp262^6.58^ (top of TM6), whereas Asp97^2.63^ (top of TM2) is of selective importance for AMD11070^48–51^. In line with these findings, our molecular docking studies suggest that AMD11070 is confined to the minor binding pocket in CXCR4, whereas plerixafor interacts with the major binding pocket. This differential binding mode allows plerixafor to stabilize a receptor conformation that engages GRKs, which in turn phosphorylates the intracellular domain to induce arrestin recruitment towards CXCR4.

We propose that the intrinsic arrestin recruitment activity of plerixafor, not mirrored in AMD11070, leads to a more efficient reversal of the CXCL12 gradient across the BM, and thereby stronger mobilization of HSCs from the BM compared to other CXCR4 antagonists (Table 1). Thus, we believe that the biased action of plerixafor leads to a synergistic effect that is central to its superior therapeutic effect on stem cell mobilization. WHIM syndrome is a congenital immunodeficiency disorder that is caused by functional overactivity of CXCR4, and manifests in e.g., retention of mature neutrophils in the BM, which in turn leads to neutropenia and increased risk of infections. While plerixafor is superior to AMD11070 in HPC mobilization (Fig. 5b) the two compounds are equipotent in neutrophil mobilization (Fig. 5c). This, together with its pharmacokinetic advantages (oral bioavailability and longer half-life) makes AMD11070 a promising candidate for treatment of WHIM, where clinical trials with AMD11070 are ongoing (ClinicalTrials.gov Identifiers: NCT03995108 and NCT03005327) and have shown promising results^67^. Theoretically, the pure antagonism of AMD11070, which will block all CXCL12-induced actions on CXCR4, could also represent a pharmacodynamic advantage over the mixed action profile of plerixafor in WHIM treatment. However, as there are numerous effects that contribute to the overall clinical outcome (Table 1), human head-to-head studies of the two compounds are required to determine their relative efficacy in WHIM. Similar head-to-head comparisons of plerixafor and AMD11070 or another full antagonist will help to determine the mechanism of action of plerixafor in the context of other diseases, that may aid in the design of future therapeutics.

**Table 1:** Effects of plerixafor vs AMD11070 on CXCR4, CXCL12, and stem cell mobilization.

	PLERIXAFOR	AMD11070
CXCR4:CXCL12 BINDING	Blocks CXCL12 binding	Blocks CXCL12 binding
CXCR4 SIGNALING AND ARRESTIN RECRUITMENT	Blocks CXCL12-mediated signaling	Blocks CXCL12-mediated signaling
	Intrinsic arrestin recruitment	No intrinsic arrestin recruitment
CXCR4 INTERNALIZATION	Allows constitutive internalization	Blocks internalization
CXCR4 SURFACE EXPRESSION	Decreases receptor expression	Increases receptor expression
CXCL12 TRANSPORT	CXCL12-transcytosis impaired	CXCL12-transcytosis maintained to some degree
CXCL12 GRADIENT	Reversal of chemokine gradient	Decreases CXCL12 bone marrow level, but does not reverse gradient
STEM CELL MOBILIZATION	Strong stem cell mobilization	Weaker mobilization

## Methods

### Tissue culture and transfection

COS-7 cells and HEK293 cells were grown at 10% CO_2_ and 37 °C in Dulbecco’s Modified Eagle’s Medium 1885 (DMEM) supplemented with 10% (v/v) fetal bovine serum (FBS), 2 mM L-glutamine, 180 units/ml penicillin, and 45 μg/ml streptomycin. The C2C12 mouse myoblast cells line was grown at 10% CO_2_ and 37 °C in DMEM 1885 containing 20% (v/v) FBS, 1% (v/v) penicillin/streptomycin, 1% (v/v) L-glutamine, 500 µg/ml hygromycin B, and 1 mg/ml G418. CHO-k1 cells were cultured at 5% CO_2_ and 37 °C in RPMI 1640 containing 10% (v/v) FBS, 180 units/ml penicillin, and 45 g/ml streptomycin. Unless otherwise stated, cells were transiently transfected using a chloroquine calcium phosphate precipitation method^68^.

HEK-293 cells, Cos-7, and CHO-k1 were bought from ATTC (cat CRL-1552, CRL-1651, CCL-61). C2C12 cells (93-0203C7) stably expressing Prolink (PK)-tagged CXCR4 and Enzyme Acceptor (EA)-tagged β-arrestin 2 were acquired from DiscoverX. Cell line authentication was guaranteed by the sources where the cells were bought. All eukaryotic cell lines were tested negative for mycoplasma on a regular basis, before and during tissue culture.

### IP accumulation assay

COS–7 cells were co-transfected with receptor construct and the chimeric G protein Gα_Δ6qi4myr_. This construct allows a G_ai_-coupled receptor to elicit IP3 turnover upon interaction with this chimeric G protein that is recognized as Gai by the receptor (at its four C-terminal amino acids) but transduces PLC activation (by the rest of the molecule)^69^. This method has previously been used to probe G_ai_-coupling of chemokine receptors, for example in CXCR3, CXCR4, CCR1, and CCR8^48,49,70–72^. One day after transfection, the cells were seeded in 24–well plates (1.5 × 10^5^ cells per well) and incubated with 2 µCi of *myo–*[^3^H]–inositol in 300 µl of growth medium for 24 h. Cells were then washed with Hanks’ buffered salt solution (HBSS) supplemented with CaCl_2_ and MgCl_2_ and afterward incubated for 15 min in 300 µl of buffer supplemented with 10 mM LiCl followed by ligand addition and 90 min of incubation. When plerixafor and AMD11070 were tested as antagonists, they were added 10 min before CXCL12. The generated [^3^H]–inositol phosphates were purified on AG 1–X8 anion exchange resins.

### β-Arrestin recruitment

Arrestin 2 recruitment was experimentally measured using the PathHunter β-arrestin assay (DiscoveRx). C2C12 cells stably express Prolink (PK)-tagged CXCR4 and Enzyme Acceptor (EA)-tagged β-arrestin 2. The cells were seeded in growth medium (20,000/well) in a poly-d-lysine coated 96-well plate and kept overnight in 5% CO_2_ at 37 °C. The cells were stimulated with various concentrations of the CXCL12, plerixafor, and AMD11070 in Opti-MEM medium with reduced serum containing GlutaMAX supplement (Gibco) and incubated for 90 min at 37 °C. The β-Arrestin 2-EA recruitment forces the complementation of the two β-galactosidase enzyme fragments (EA and PK) and allows to quantify β-arrestin recruitment by replacing the medium with PathHunter detection reagent solution [Galacton Star (1:25), Emerald II (5:25) and cell assay buffer (19:25); DiscoveRx]. After 1 h of incubation in the dark, luminescence was measured using the EnVision Multilabel Reader (PerkinElmer).

### Antibody-feeding internalization assay

HEK293 cells were seeded and transfected with CXCR4 or US28 constructs. 48 hours after transfection, the cells were incubated in cold DMEM medium containing mouse M1 anti-FLAG antibody (Sigma-Aldrich) at 2 μg/mL for 1 h at 4 °C. No CaCl_2_ was added as DMEM contains 1.8 mM CaCl_2_. After three washes in cold DMEM, the specimens were either immediately fixed (*t* = 0) in 4% paraformaldehyde for 20 min or incubated in prewarmed DMEM media at 37 °C for 30 min to induce internalization and then fixed (*t* = 30). Furthermore, the CXCR4 agonist CXCL12 was included at 1 μM to induce agonist-mediated internalization of this receptor. Following three washes in TBS, the coverslips were blocked in TBS with 2% BSA for 20 min. Subsequently, they were incubated with goat anti-mouse Alexa-Fluor 488-conjugated IgG antibody (Molecular Probes) diluted 1:1000 in TBS containing 1% BSA for 30 min to specifically detect labeled receptors residing at the cell surface. After three washes in TBS, the specimens were permeabilized in TBS with 1% BSA and 0.2% saponin for 20 min. Following, the coverslips were incubated with goat anti-mouse Alexa-Fluor 568- conjugated IgG antibody (Molecular Probes) diluted 1:1000 in TBS containing 1% BSA and 0.2% saponin for 30 min to detect labeled internalized receptors. The specimens were finally mounted in SlowFade Antifade reagent (Molecular Probes) using nail polish as sealing after three washes. The experiment was performed thrice. Except for the first step all the others were performed at room temperature. Mock transfected cells were included to ensure no unspecific binding of the antibodies.

### Time-course ELISA-based internalization assay

HEK293 cells were seeded and transiently transfected with FLAG-tagged CXCR4 and US28 at 15 ng/well. 48 hours after transfection, the cells were incubated in cold DMEM containing mouse M1 anti-FLAG antibody at 2 μg/mL (Sigma-Aldrich) for 1 h at 4 °C. After three washes in cold DMEM, the cells were either immediately fixed (*t* = 0) or incubated in pre-warmed DMEM media at 37 °C for various time-periods (*t* = 1, 5, 10, 20, and 30 min) to induce internalization and subsequently fixed. From here on, the procedure followed that of the standard ELISA assay. The results are presented as the amount of ELISA signal at a given time-point relative to that at *t* = 0. The experiment was performed twice in quadruples. Absorbance was measured at 450 nm on an EnVision Multilabel Reader (Perkin Elmer).

### Receptor surface expression by ELISA

CHO–k1 cells were transfected with FLAG-tagged CXCR4. One day after transfection, the cells were seeded in 96–well plates (4 × 10^4^ cells per well) and incubated with ligand in growth medium overnight. Cells were washed in Tris-buffered saline (TBS) and then fixated with 3.7% (v/v) formaldehyde for 10 min at RT. After fixation, cells were washed and incubated in a blocking solution (TBS with 2% (w/v) BSA) for 30 min. Cells were then incubated for 1.5 h with anti–FLAG antibody (Sigma–Aldrich) at 2 μg ml^–1^ in TBS with 1 mM CaCl_2_ and 1% (w/v) BSA. After washing with TBS/CaCl_2_/BSA, the cells were incubated with goat anti-mouse HRP–conjugated antibody (Abcam) at a 1:1000 dilution (v/v). Following additional washing, the immunoreactivity was revealed by the addition of TMB Plus substrate (Kem–En–Tec), and the reaction was stopped with 0.2 M H_2_SO_4_ after 5 min. Absorbance was measured at 450 nm on a 2104 EnVision Multilabel Reader (Perkin Elmer).

### SNAP-tagged receptor internalization

Real-time internalization assays were performed as previously published^73,74^. HEK293A wild-type cells transiently expressing the SNAP-tagged CXCR4 using the lipofectamine transfection system were seeded in white 384-well plates the day after transfection at a density of 2 × 10^4^ cells/well. The following day, the media was removed, and the SNAP-tagged receptors were labeled with 100 nM Tag-lite SNAP-Lumi4-Tb (donor) in OptiMEM for 60 min at 37 °C. Subsequently, the cells were washed with HBBS supplemented with 1 mM CaCl_2_, 1 mM MgCl_2_, 20 mM HEPES (internalization buffer, pH 7.4). Hereafter, 100 μM preheated fluorescein (acceptor) was added. The plate was placed in a 37 °C incubator for 5 min prior to ligand addition to adjust the temperature. The cells were stimulated with 37 °C preheated CXCL12, plerixafor, and AMD11070, and internalization was measured every 6 min at 37 °C an EnVision Multilabel Reader (Perkin Elmer) by measuring emission at 520 nm and 615 nm after excitation at 340 nm. Receptor internalization was calculated as the ratio 615/520 nm.

### Arrestin recruitment by Bioluminescence Resonance Energy Transfer assay (BRET)

CHO cells were transiently transfected with CXCR3 wt or CXCR3-[K300A, S304E] and the BRET donor Rluc8-Arrestin-3-Sp1 and BRET acceptor mem-linker-citrine-SH3 using the lipofectamine based transfection method. One day after transfection, cells were washed in PBS and resuspended in PBS with 5 mmol/L glucose. Then 85 µl of cell suspension solution was added per well of a 96-well plate followed by the addition of PBS with coelenterazine-h 5 µmol/L. Following a 10 min preincubation, increasing concentrations of ligand were added and incubated for an additional 40 min. Luminescence was measured by an EnVision Multilabel Reader (Perkin Elmer) and signaling calculated as the BRET ratio Rluc8-485nm/YFP-530nm.

### In vivo studies

C57Bl/6 J female mice between 6–8 weeks old were used in all the experiments. All mice were housed in specific pathogen-free conditions at Imperial College London. All experiments were carried out in accordance with the recommendations in the Guide for the Use of Laboratory Animals of Imperial College London. All animal procedures and care conformed strictly to the UK Home Office Guidelines under the Animals (Scientific Procedures) Act 1986, and the protocols were approved by the Home Office of Great Britain.

Mice were i.p. injected with plerixafor (5 mg/kg, Sigma-Aldrich), AMD11070 (5 mg/kg), or PBS as a control in a total volume of 400 μl for 1 h. Mice were then euthanized via an overdose of pentobarbital and blood was collected in EDTA coated syringes by cardiac puncture. Erythrocyte lysis of the blood was carried out and samples were centrifuged at 450 *g* for 5 min at 4˚C. Bone marrow was collected by flushing a femur and, the spleens were homogenized.

### CFU-HPC assays

To grow CFU-HPCs colonies we used MethoCult^TM^ complete medium (M3434, StemCell Technologies^TM^). Bone marrow cells were plated at 2 × 10^4^, spleen and blood cells at 1 × 10^5^ for 12 days in incubators at 37 °C, 5% CO_2_. Colonies were counted under a bright field microscope.

### Flow cytometry

To count the total number of neutrophils, single-cell suspensions from blood, bone marrow, and spleen were stained live/dead near-IR stain (Life Technologies) and Fc-receptors block was performed (using clone 93, BioLegend). Cell suspensions were incubated with directly conjugated fluorescent antibodies for 10 min at room temperature. The following Abs were used: Ly6G (clone 1A8), CD45 (clone 30-F11), CD11b (clone M1/70), CD3e (clone 17 A2), CD19 (clone 6D5), Ter119 (clone TER-119), CD62L (clone MEL-14), CXCR4 (clone 2B11). Fluorescence minus one (FMO) controls were used to validate each directly conjugated fluorescent antibody. The acquisition was performed on BDFortessa using FacsDiva software (BD Bioscience) with further analysis by FlowJo software.

### ELISA

Supernatant from bone marrow, spleen, and serum from blood was used to test CXCL12 by ELISA (R&D Systems, DY460).

### Molecular modeling

The Schrödinger Small-Molecule Drug Discovery Suite (release 2019-3)^75^ was used for all molecular modeling procedures, using the OPLS3e force field. If not specified otherwise, the default settings were used.

Preparation of the receptor structure: The full-length model of human CXCR4 in complex with its natural ligand CXCL12^53^ was imported and prepared with the Protein Preparation Wizard workflow. Default settings were used in the first preparation step (*pre-process*), and in the next step (*review/modify*) the CXCL12 ligand was deleted to give the apo structure of CXCR4. In the final step (*refine*), an H-bond assignment (prediction of protonation states (pH 7.4) and reorientation of functional groups) was performed to optimize H-bond networks, followed by a restrained minimization (root mean square deviation of 0.30 Å for heavy atoms) to relieve strain.

Preparation of ligand structures: plerixafor contains eight amino groups, and at pH 7.4 this compound was predicted to have a total charge of 4 + (LigPrep), which was distributed in 6 different ways (protonation states). Moreover, as the input conformation of the two bicyclam rings is not changed during docking, 64 different ring conformations were generated (ConfGen) for each of the 6 protonation states. The 10 conformations with the lowest energy for each protonation state were selected to give a total of 6 × 10 = 60 input ligand structures of plerixafor for docking.

(*S*)-AMD11070 contains a primary and a tertiary amino group and was predicted to have a total charge of either 1 + (protonated primary amine) or 2 + (protonated primary and tertiary amine) at pH 7.4. As the software treats the tertiary amine as an additional stereocenter (not allowed to invert during docking), both configurations of this “stereocenter” had to be included to give a total of 2 × 2 = 4 input ligand structures of AMD11070 for docking.

Docking: The standard protocol of the induced fit docking workflow was used for the docking of both compounds to the CXCR4 structure. To position the docking box approximately in the middle of the binding pocket, it was centered on residues Y116/D171/Y255/E288. The trim option (temporary mutation to Ala) was used to avoid steric conflict with the long and flexible Arg188 side chain, which protruded from ECL2 down into the main binding pocket. In all cases, the top 20 poses within an energy window of 30 kcal/mol were kept for analysis.

For plerixafor (60 different input ligand structures) H-bond constraints were put on D171 and E288, and the standard precision (SP) option was used for the final redocking step. This resulted in a total of 132 poses, where the top-scoring pose had a docking score of −10.1 kcal/mol. The 132 poses were manually inspected for additional favorable interactions with D262, which is known to be important for the binding and function of plerixafor, resulting in the identification of the binding mode for plerixafor that is shown in Fig. 4 and discussed in the text.

For AMD11070 (4 different input ligand structures) an H-bond constraint was placed on E288, and the extra precision (XP) option was chosen for the final redocking step. This resulted in a total of 45 poses; the binding mode for AMD11070 that is presented in the text (Fig. 4) was the top-scoring pose with a docking score of −11.5 kcal/mol.

### Statistics and reproducibility

All calculations and statistical analysis were performed using GraphPad Prism (GraphPad Software, Inc). For in vivo studies all experiment represents *n* = 4–5 and statistical significance was analyzed using 1way ANOVA with a Tukey’s multiple comparison post-test. Exact *p*-values are shown on respective graphs. Sigmoid dose–response curves and IC_50_/E_C50_ values were determined by nonlinear regression with a logistically fit of data from at least three independent experiments. Real-time receptor internalization and halftimes were analyzed using one-phase association non-linear regression and represent data from three or five independent experiments. All experiments were run in duplicates or triplicates as technical replicates as indicated in figure legends.

### Reporting summary

Further information on research design is available in the Nature Research Reporting Summary linked to this article.

## Supplementary information

Supplementary Information

Description of Additional Supplementary Files

Supplementary Data 1

Supplementary Data 2

Reporting Summary

## Data Availability

Data that support the findings of this study are available from the corresponding author upon reasonable request. Source data for all graphs and chart are provided with the paper as supplementary data.

## References

[1] Hauser AS, Attwood MM, Rask-Andersen M, Schiöth HB, Gloriam DE (2017). Trends in GPCR drug discovery: new agents, targets and indications. Nat. Rev. Drug Discov..

[2] Marchese A (2003). The E3 ubiquitin ligase AIP4 mediates ubiquitination and sorting of the G protein-coupled receptor CXCR4. Dev. Cell.

[3] Gurevich VV, Gurevich EV (2019). GPCR signaling regulation: the role of GRKs and arrestins. Front. Pharmacol..

[4] Kenakin, T. The Application of Signaling Bias to New Therapeutic Drug Therapy for Seven Transmembrane (G Protein-coupled) Receptors: Quantifying Bias in Biased Signaling in Physiology, Pharmacology and Therapeutics (ed. Arey, J. B.) 81–102 (Elsevier, 2014).

[5] Murphy PM (2000). International union of pharmacology. XXII. Nomencl. Chemokine Recept. Pharmacol. Rev..

[6] Feng Y, Broder CC, Kennedy PE, Berger EA (1996). HIV-1 entry cofactor: functional cDNA cloning of a seven-transmembrane, G protein-coupled receptor. Science.

[7] Nagasawa T (1996). Defects of B-cell lymphopoiesis and bone-marrow myelopoiesis in mice lacking the CXC chemokine PBSF/SDF-1. Nature.

[8] McGrath KE, Koniski AD, Maltby KM, McGann JK, Palis J (1999). Embryonic expression and function of the chemokine SDF-1 and its receptor, CXCR4. Dev. Biol..

[9] Möhle R (1998). The chemokine receptor CXCR-4 is expressed on CD34+ hematopoietic progenitors and leukemic cells and mediates transendothelial migration induced by stromal cell-derived factor-1. Blood.

[10] Dar A, Kollet O, Lapidot T (2006). Mutual, reciprocal SDF-1/CXCR4 interactions between hematopoietic and bone marrow stromal cells regulate human stem cell migration and development in NOD/SCID chimeric mice. Exp. Hematol..

[11] Liao YX (2013). The role of the CXCL12-CXCR4/CXCR7 axis in the progression and metastasis of bone sarcomas (Review). Int. J. Mol. Med..

[12] Dewan MZ (2006). Stromal cell-derived factor-1 and CXCR4 receptor interaction in tumor growth and metastasis of breast cancer. Biomed. Pharmacother..

[13] Huang CY (2009). Stromal cell-derived factor-1/CXCR4 enhanced motility of human osteosarcoma cells involves MEK1/2, ERK and NF-κB-dependent pathways. J. Cell. Physiol..

[14] Kim, S. Y. et al. Inhibition of the CXCR4/CXCL12 chemokine pathway reduces the development of murine pulmonary metastases. *Clin. Exp. Metastasis*. 10.1007/s10585-007-9133-3 (2007). 10.1007/s10585-007-9133-3 PMC2730112 18071913

[15] Hendrix CW (2000). Pharmacokinetics and safety of AMD-3100, a novel antagonist of the CXCR- 4 chemokine receptor, in human volunteers. Antimicrob. Agents Chemother..

[16] De Clercq E (2019). Mozobil^®^ (Plerixafor, AMD3100), 10 years after its approval by the US Food and Drug Administration. Antivir. Chem. Chemother..

[17] Debnath B, Xu S, Grande F, Garofalo A, Neamati N (2013). Small molecule inhibitors of CXCR4. Theranostics.

[18] Kazmierski WM, Gudmundsson KS, Piscitelli SC (2007). Small molecule CCR5 and CXCR4-based viral entry inhibitors for anti-HIV therapy currently in development. Annu. Rep. Med. Chem..

[19] Moyle G (2009). Proof of activity with AMD11070, an orally bioavailable inhibitor of CXCR4‐tropic HIV type 1. Clin. Infect. Dis..

[20] Mosi RM (2012). The molecular pharmacology of AMD11070: an orally bioavailable CXCR4 HIV entry inhibitor. Biochem. Pharmacol..

[21] Stone ND (2007). Multiple-dose escalation study of the safety, pharmacokinetics, and biologic activity of oral AMD070, a selective CXCR4 receptor inhibitor, in human subjects. Antimicrob. Agents Chemother..

[22] Berg C (2018). Inhibition of HIV fusion by small molecule agonists through efficacy-engineering of CXCR4. ACS Chem. Biol..

[23] O’Boyle G (2013). Inhibition of CXCR4-CXCL12 chemotaxis in melanoma by AMD11070. Br. J. Cancer.

[24] Redpath AN, François M, Wong SP, Bonnet D, Rankin SM (2017). Two distinct CXCR4 antagonists mobilize progenitor cells in mice by different mechanisms. Blood Adv..

[25] Dar A (2005). Chemokine receptor CXCR4-dependent internalization and resecretion of functional chemokine SDF-1 by bone marrow endothelial and stromal cells. Nat. Immunol..

[26] Dar A (2011). Rapid mobilization of hematopoietic progenitors by AMD3100 and catecholamines is mediated by CXCR4-dependent SDF-1 release from bone marrow stromal cells. Leukemia.

[27] Pillay J (2020). Effect of the CXCR4 antagonist plerixafor on endogenous neutrophil dynamics in the bone marrow, lung and spleen. J. Leukoc. Biol..

[28] Signoret N (1997). Phorbol esters and SDF-1 induce rapid endocytosis and down modulation of the chemokine receptor CXCR4. J. Cell Biol..

[29] Alkhatib G, Locati M, Kennedy PE, Murphy PM, Berger EA (1997). HIV-1 coreceptor activity of CCR5 and its inhibition by chemokines: independence from G protein signaling and importance of corecepter downmodulation. Virology.

[30] Alkhatib G (2009). The biology of CCR5 and CXCR4. Curr. Opin. HIV AIDS.

[31] Amara A (1997). HIV coreceptor downregulation as antiviral principle: SDF-1α-dependent internalization of the chemokine receptor CXCR4 contributes to inhibition of HIV replication. J. Exp. Med..

[32] Signoret N (1998). Differential regulation of CXCR4 and CCR5 endocytosis. J. Cell Sci..

[33] Orsini MJ, Parent JL, Mundell SJ, Benovic JL (1999). Trafficking of the HIV coreceptor CXCR4. Role of arrestins and identification of residues in the C-terminal tail that mediate receptor internalization. J. Biol. Chem..

[34] Haribabu B (1997). Regulation of human chemokine receptors CXCR4: role of phosphorylation in desensitization and internalization. J. Biol. Chem..

[35] Tarasova NI, Stauber RH, Michejda CJ (1998). Spontaneous and ligand-induced trafficking of CXC-chemokine receptor 4. J. Biol. Chem..

[36] Marchese A, Benovic JL (2001). Agonist-promoted ubiquitination of the G protein-coupled receptor CXCR4 mediates lysosomal sorting. J. Biol. Chem..

[37] Fraile-Ramos A (2001). The human cytomegalovirus US28 protein is located in endocytic vesicles and undergoes constitutive endocytosis and recycling. Mol. Biol. Cell.

[38] Zastrows, M. Von & Kobilkasofl, B. K. Antagonist-dependent and -independent steps in the mechanism of adrenergic receptor internalization. *Mol. Biol. Cell* **269**, 18448–18452 (1994). 7518433

[39] Katakam, P. V. G. et al. Enhanced endothelin-1 response and receptor expression in small mesenteric arteries of insulin-resistant rats. *Am. J. Physiol. Heart. Circ. Physiol.* **27157**, 522–527 (2020). 10.1152/ajpheart.2001.280.2.H522 11158947

[40] Janssens R (2017). Truncation of CXCL12 by CD26 reduces its CXC chemokine receptor 4- and atypical chemokine receptor 3-dependent activity on endothelial cells and lymphocytes. Biochem. Pharmacol..

[41] Sun Y, Cheng Z, Ma L, Pei G (2002). β-arrestin2 is critically involved in CXCR4-mediated chemotaxis, and this is mediated by its enhancement of p38 MAPK activation. J. Biol. Chem..

[42] Fong AM (2002). Defective lymphocyte chemotaxis in β-arrestin2- and GRK6-deficient mice. Proc. Natl Acad. Sci. U.S.A..

[43] Sotsios Y, Ward SG (2000). Phosphoinositide 3-kinase: a key biochemical signal for cell migration in response to chemokines. Immunol. Rev..

[44] Song Q, Ji Q, Li Q (2018). The role and mechanism of β-arrestins in cancer invasion and metastasis (Review). Int. J. Mol. Med..

[45] Janssens R (2018). Peroxynitrite exposure of CXCL12 impairs monocyte, lymphocyte and endothelial cell chemotaxis, lymphocyte extravasation in vivo and anti-HIV-1 activity. Front. Immunol..

[46] Hitchinson, B. et al. Biased antagonism of CXCR4 avoids antagonist tolerance. *Sci. Signal*. **11**, eaat2214 (2018). 10.1126/scisignal.aat2214 PMC6422681 30327409

[47] Gerlach LO (2003). Metal ion enhanced binding of AMD3100 to Asp262 in the CXCR4 receptor. Biochemistry.

[48] Rosenkilde MM (2004). Molecular mechanism of AMD3100 antagonism in the CXCR4 receptor: transfer of binding site to the CXCR3 receptor. J. Biol. Chem..

[49] Rosenkilde MM (2007). Molecular mechanism of action of monocyclam versus bicyclam non-peptide antagonists in the CXCR4 chemokine receptor. J. Biol. Chem..

[50] Wong RSY (2008). Comparison of the potential multiple binding modes of bicyclam, monocylam, and noncyclam small-molecule CXC chemokine receptor 4 inhibitors. Mol. Pharmacol..

[51] Gerlach LO, Skerlj RT, Bridger GJ, Schwartz TW (2001). Molecular interactions of cyclam and bicyclam non-peptide antagonists with the CXCR4 chemokine receptor. J. Biol. Chem..

[52] Ballesteros JA, Weinstein H (1995). Integrated methods for the construction of three-dimensional models and computational probing of structure-function relations in G protein-coupled receptors. Methods Neurosci..

[53] Ngo T (2020). Crosslinking-guided geometry of a complete CXC receptor-chemokine complex and the basis of chemokine subfamily selectivity. PLoS Biol..

[54] Induced Fit Docking protocol; Glide, Schrödinger, LLC, New York, NY, (2016); Prime, Schrödinger, LLC, New York, NY. (2019).

[55] Wu B (2010). Structures of the CXCR4 chemokine GPCR with small-molecule and cyclic peptide antagonists. Science.

[56] Kufareva I, Gustavsson M, Zheng Y, Stephens BS, Handel TM (2017). What do structures tell us about chemokine receptor function and antagonism?. Annu. Rev. Biophys..

[57] Rosenkilde M, Schwartz T (2012). GluVII:06—a highly conserved and selective anchor point for non-peptide ligands in chemokine receptors. Curr. Top. Med. Chem..

[58] Weis WI, Kobilka BK (2018). The molecular basis of G protein–coupled receptor activation. Annu. Rev. Biochem..

[59] Zhou XE (2017). Identification of phosphorylation codes for arrestin recruitment by G protein-coupled receptors. Cell.

[60] Kang Y (2015). Crystal structure of rhodopsin bound to arrestin by femtosecond X-ray laser. Nature.

[61] Staus DP (2020). Structure of the M2 muscarinic receptor–β-arrestin complex in a lipid nanodisc. Nature.

[62] Huang W (2020). Structure of the neurotensin receptor 1 in complex with β-arrestin 1. Nature.

[63] Evrard M (2018). Developmental analysis of bone marrow neutrophils reveals populations specialized in expansion, trafficking, and effector functions. Immunity.

[64] Hattori K (2001). Plasma elevation of stromal cell-derived factor-1 induces mobilization of mature and immature hematopoietic progenitor and stem cells. Blood.

[65] De Filippo K, Rankin SM (2018). CXCR4, the master regulator of neutrophil trafficking in homeostasis and disease. Eur. J. Clin. Investig..

[66] Hatse S, Princen K, Bridger G, De Clercq E, Schols D (2002). Chemokine receptor inhibition by AMD3100 is strictly confined to CXCR4. FEBS Lett..

[67] Dale, D. C. et al. Results of a phase 2 trial of an oral CXCR4 antagonist mavorixafor for treatment of WHIM syndrome. *Blood*. 10.1182/blood.2020007197 (2020). 10.1182/blood.2020007197 PMC7770568 32870250

[68] Kissow H (2012). Glucagon-like peptide-1 (GLP-1) receptor agonism or DPP-4 inhibition does not accelerate neoplasia in carcinogen treated mice. Regul. Pept..

[69] Kostenis, E., Zeng, F. & Wess, J. Functional characterization of a series of mutant G protein αq subunits displaying promiscuous receptor coupling properties. *J. Biol. Chem.* **273**, 17886–17892 (1998). 10.1074/jbc.273.28.17886 9651394

[70] Rosenkilde MM, Andersen MB, Nygaard R, Frimurer TM, Schwartz TW (2007). Activation of the CXCR3 chemokine receptor through anchoring of a small molecule chelator ligand between TM-III, -IV, and -VI. Mol. Pharmacol..

[71] Jensen PC, Thiele S, Ulven T, Schwartz TW, Rosenkilde MM (2008). Positive versus negative modulation of different endogenous chemokines for CC-chemokine receptor 1 by small molecule agonists through allosteric versus orthosteric binding. J. Biol. Chem..

[72] Jensen PC (2007). Molecular interaction of a potent nonpeptide agonist with the chemokine receptor CCR8. Mol. Pharmacol..

[73] Roed SN (2014). Real-time trafficking and signaling of the glucagon-like peptide-1 receptor. Mol. Cell. Endocrinol..

[74] Foster, S. R. & Bräuner-Osborne, H. Investigating Internalization and Intracellular Trafficking of GPCRs: New Techniques and Real-Time Experimental Approaches in Targeting Trafficking in Drug Development. *Handbook of Experimental Pharmacology* (eds. Ulloa-Aguirre, A. & Tao, Y. X.) 41–61 (Springer, 2017). 10.1007/164_2017_57 29018878

[75] Schrödinger, LLC, New York, NY. (2019).

[76] Zhang J, Yang J, Jang R, Zhang Y (2015). GPCR-I-TASSER: a hybrid approach to G protein-coupled receptor structure modeling and the application to the human genome. Structure.

