# Supplementary material for: Biased action of the CXCR4-targeting drug plerixafor is essential for its superior hematopoietic stem cell mobilization
Source: Commun Biol. 2021 May 12;4:569. doi: 10.1038/s42003-021-02070-9 (PMC8115334; doi:10.1038/s42003-021-02070-9)
Supplement: Supplementary file 1 — Supplementary Information [file 42003_2021_2070_MOESM1_ESM.pdf]

Supplementary material

Biased action of the CXCR4-targeting drug plerixafor is essential for its superior hematopoietic stem cell mobilization

Astrid S. Jørgensen[1]#, Viktorija Daugvilaite[1]#, Katia De Filippo[2], Christian Berg[1,3], Masa Mavri[1,4], Tau Benned-Jensen[1,§], Goda Juzenaite[2], Gertrud Hjortø[1], Sara Rankin[2], Jon Våbenø[5]*, Mette M Rosenkilde[1]*

[1]Department of Biomedical Sciences, Faculty of Health and Medical Sciences, The Panum Institute, University of Copenhagen, Copenhagen, Denmark
[2]Department of Medicine, National Heart and Lung Institute (NHLI), Imperial College, London, United Kingdom
[3]Unit for Infectious Diseases, Department of Medicine, Herlev-Gentofte Hospital, University of Copenhagen, Herlev, Denmark.
[4]Institute of Preclinical Sciences, Veterinary Faculty, University of Ljubljana, Ljubljana, Slovenia
[5]Helgeland Hospital Trust, Prestmarkveien 1, 8800 Sandnessjøen, Norway
§ Present address: Lundbeck A/S, Ottiliavej 9, 2500 Valby, Copenhagen, Denmark
# These authors contributed equally
* These authors jointly supervised this work

Table of content:

Supplementary figure 1, p.2

Supplementary figure 2, p.3

Supplementary figure 3, p.4

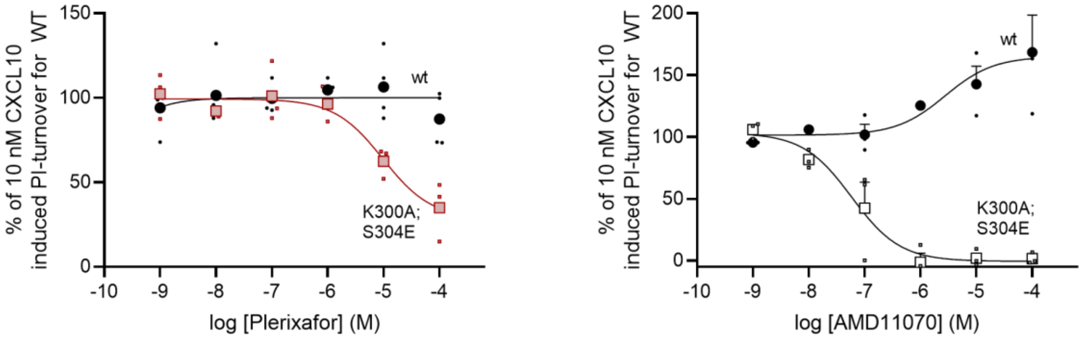

Supplementary Figure 1: Transferring of plerixafor binding site to CXCR3 reveals partial agonism.

Antagonistic effect of plerixafor and AMD11070 on CXCL10-induced CXCR3 G protein activity. The receptor activity was measured by inositol triphosphate (IP3) accumulation in H EK293 cells transiently transfected with WT CXCR3 (black circle) or CXCR3[K300A-S 304E] (squares) and the chimeric G protein Gqi4myr. Cells were stimulated with 10nM CXCL10 before the addition of plerixafor (red) or AMD10070 (white). Data for plerixafor pr eviously published in [48]. Data is normalized to the signal by 10nM CXCL10 alone and sho wn with mean ± SEM of duplicates from at least three independent experiments.

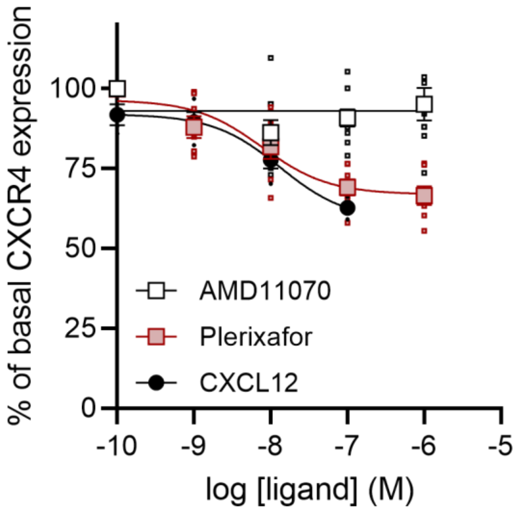

Supplementary Figure 2: Plerixafor and AMD11070 differentially affect CXCR4 surface expression after chronic exposure.

CXCR4 cell surface expression, determined by ELISA, after chronic exposure to CXCL12, plerixafor, or AMD11070. CHO-K1 cells transfected with FLAG-tagged CXCR4 were incubated with ligands overnight before wash and staining. Data were normalized to the CXCR4 levels in unstimulated cells and shown with % mean ± SEM of duplicates from at least four independent experiments. CXCL12 (black circles), plerixafor (red squares) and AMD11070 (white squares).

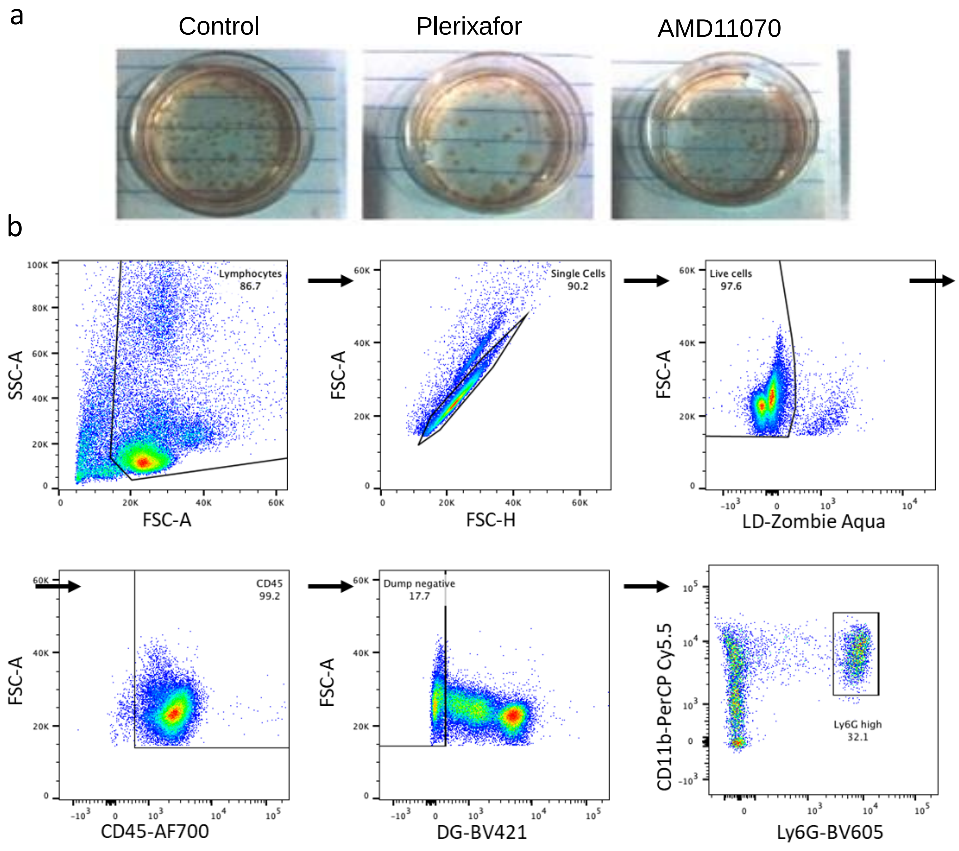

Supplementary Figure 3: *In vivo* effect of plerixafor and AMD11070 on HPC from the BM.

a) Photos of colonies from BM of control (PBS), plerixafor, and AMD11070 treated mice. Plerixafor has fewer colonies compared with both control and AMD11070. This is one representation of 5 replications. **b**) Flow cytometry gating strategy to quantify numbers of neutrophils. An example of the gating strategy on murine blood samples is shown but represents the general gating strategy applied to all samples. Neutrophils were detected as Ly6G-positive, CD11b-positive lymphocytes. Lymphocytes were selected based on FSC and SSC following selection of single cells and gated on Zombie Aqua negative, following selection of CD45-positive population. A dump channel was used to remove CD3e, CD19, and Ter119 positive cells using the dump gate. 1A8 clone against Ly6G and M1/70 clone against CD11b were used to identify neutrophils.
