# Supplementary material for: Biased action of the CXCR4-targeting drug plerixafor is essential for its superior hematopoietic stem cell mobilization
Source: Commun Biol. 2021 May 12;4:569. doi: 10.1038/s42003-021-02070-9 (PMC8115334; doi:10.1038/s42003-021-02070-9)
Supplement: Supplementary file 2 — Description of Additional Supplementary Files [file 42003_2021_2070_MOESM2_ESM.pdf]

Description of Additional Supplementary Files

File name: Supplementary Data 1

Description: The table is a non-exhaustive list of clinical trials where plerixafor has been tested for different clinical indications. Plerixafor is used under several drug-names; AMD3100, LM2987, JM 3100, Plerixafor, Mozobil.

File name: Supplementary Data 2

Description: Source data for all graphs and chart.
