# Supplementary material for: Biased action of the CXCR4-targeting drug plerixafor is essential for its superior hematopoietic stem cell mobilization
Source: Commun Biol. 2021 May 12;4:569. doi: 10.1038/s42003-021-02070-9 (PMC8115334; doi:10.1038/s42003-021-02070-9)
Supplement: Supplementary file 5 — Reporting Summary [file 42003_2021_2070_MOESM5_ESM.pdf]

nature research

Corresponding author(s): Mette Rosenkilde and Jon Våbenø

Last updated by author(s): Mar 15, 2021

Reporting Summary

Nature Research wishes to improve the reproducibility of the work that we publish. This form provides structure for consistency and transparency in reporting. For further information on Nature Research policies, see our Editorial Policies and the Editorial Policy Checklist.

Statistics

For all statistical analyses, confirm that the following items are present in the figure legend, table legend, main text, or Methods section.

n/a	Confirmed	
☐	☒	The exact sample size (*n*) for each experimental group/condition, given as a discrete number and unit of measurement
☐	☒	A statement on whether measurements were taken from distinct samples or whether the same sample was measured repeatedly
☐	☒	The statistical test(s) used AND whether they are one- or two-sided<br>*Only common tests should be described solely by name; describe more complex techniques in the Methods section.*
☒	☐	A description of all covariates tested
☐	☒	A description of any assumptions or corrections, such as tests of normality and adjustment for multiple comparisons
☐	☒	A full description of the statistical parameters including central tendency (e.g. means) or other basic estimates (e.g. regression coefficient) AND variation (e.g. standard deviation) or associated estimates of uncertainty (e.g. confidence intervals)
☒	☐	For null hypothesis testing, the test statistic (e.g. *F*, *t*, *r*) with confidence intervals, effect sizes, degrees of freedom and *P* value noted<br>*Give P values as exact values whenever suitable.*
☒	☐	For Bayesian analysis, information on the choice of priors and Markov chain Monte Carlo settings
☒	☐	For hierarchical and complex designs, identification of the appropriate level for tests and full reporting of outcomes
☒	☐	Estimates of effect sizes (e.g. Cohen's *d*, Pearson's *r*), indicating how they were calculated

Our web collection on statistics for biologists contains articles on many of the points above.

Software and code

Policy information about availability of computer code

Data collection	Data was collected as described in materials and methods section
Data analysis	Prism8 was used for analyzing the data from the functional signaling experiment and analysis of in vivo studies.<br>The Schrödinger Small-Molecule Drug Discovery Suite (release 2019-3) was used for all molecular modelling procedures, using the OPLS3e force field

For manuscripts utilizing custom algorithms or software that are central to the research but not yet described in published literature, software must be made available to editors and reviewers. We strongly encourage code deposition in a community repository (e.g. GitHub). See the Nature Research guidelines for submitting code & software for further information.

Data

Policy information about availability of data

All manuscripts must include a data availability statement. This statement should provide the following information, where applicable:

- Accession codes, unique identifiers, or web links for publicly available datasets
- A list of figures that have associated raw data
- A description of any restrictions on data availability

The data that support the findings of this study are available from the corresponding
author upon reasonable request.Source data for all graphs and chart are provided with the paper as supplementary data.

Field-specific reporting

Please select the one below that is the best fit for your research. If you are not sure, read the appropriate sections before making your selection.

- [x] Life sciences
- [ ] Behavioural & social sciences
- [ ] Ecological, evolutionary & environmental sciences

For a reference copy of the document with all sections, see nature.com/documents/nr-reporting-summary-flat.pdf

Life sciences study design

All studies must disclose on these points even when the disclosure is negative.

Sample size	tested in parallel with buffer in dublicates and triplicates at least 3 times, excact number of replicates in figure legends<br>The variance of the results is presented as standard error of the mean in the sum curves of three or more individual biological replicates
Data exclusions	Experiments were excluded if the controls indicated experimental problems e.g. negative data for the positive controls. Data points were excluded when there was a technical mistake in the ligand preparation or during the procedure of the experiment.
Replication	The ligands have been tested in at least three individual biological replicates and the preparation of the dilutions rows of the ligands for each type of experiment has been performed at least two times, to avoid mistakes.
Randomization	It was not necessary to randomize samples in the experiments. As the experiments were performed by individual researchers it was not possible to blind within the different experiments.
Blinding	Investigators were not blinded as this was not needed for the studies

Reporting for specific materials, systems and methods

We require information from authors about some types of materials, experimental systems and methods used in many studies. Here, indicate whether each material, system or method listed is relevant to your study. If you are not sure if a list item applies to your research, read the appropriate section before selecting a response.

Materials & experimental systems

n/a	Involved in the study	
[ ]	[x]	Antibodies
[ ]	[x]	Eukaryotic cell lines
[x]	[ ]	Palaeontology and archaeology
[ ]	[x]	Animals and other organisms
[x]	[ ]	Human research participants
[x]	[ ]	Clinical data
[x]	[ ]	Dual use research of concern

Methods

n/a	Involved in the study	
[x]	[ ]	ChIP-seq
[ ]	[x]	Flow cytometry
[x]	[ ]	MRI-based neuroimaging

Antibodies

Antibodies used	To count total number of neutrophils, single cell suspensions from blood, bone marrow and spleen were stained Live/Dead near-IR stain (Life Technologies) and Fc-Receptors block was performed (using clone 93, BioLegend). Cell suspensions were incubated with directly conjugated fluorescent antibodies for 10 min at room-temperature. The following Abs were used: Ly6G (clone 1A8), CD45 (clone 30-F11), CD11b (clone M1/70), CD3e (clone 17 A2), CD19 (clone 6D5), Ter119 (clone TER-119), CD62L (clone MEL-14), CXCR4 (clone 2B11).
Validation	Fluorescence minus one (FMO) controls was used to validate each directly conjugated fluorescent antibody

Eukaryotic cell lines

Policy information about cell lines

Cell line source(s)	HEK-293 cells, Cos-7, and CHO-k1 were bought from ATTC (cat CRL-1552, CRL-1651, CCL-61)<br>C2C12 cells (93-0203C7) stably express Prolink (PK)-tagged CXCR4 and Enzyme Acceptor (EA)-tagged β-arrestin 2 were aqcuired from DiscoverX.
Authentication	Cell line authentication was guaranteed by the sources were the cells were bought.
Mycoplasma contamination	All eukaryotic cell lines were tested negative for mycoplasma on a regular basis, before and during tissue culture.

Commonly misidentified lines (See ICLAC register)	The study did not involve any commonly misidentified lines

Animals and other organisms

Policy information about studies involving animals; ARRIVE guidelines recommended for reporting animal research

Laboratory animals	C57Bl/6J female mice between 6-8 weeks old were used in all the experiments
Wild animals	The study did not involve wild animals
Field-collected samples	The study did not involve field-collected samples
Ethics oversight	All mice were housed in specific pathogen free conditions at Imperial College London. All experiments were carried out in accordance with the recommendations in the Guide for the Use of Laboratory Animals of Imperial College London. All animal procedures and care conformed strictly to the UK Home Office Guidelines under the Animals (Scientific Procedures) Act 1986, and the protocols were approved by the Home Office of Great Britain.

Note that full information on the approval of the study protocol must also be provided in the manuscript.

Flow Cytometry

Plots

Confirm that:

- [x] The axis labels state the marker and fluorochrome used (e.g. CD4-FITC).
- [x] The axis scales are clearly visible. Include numbers along axes only for bottom left plot of group (a 'group' is an analysis of identical markers).
- [x] All plots are contour plots with outliers or pseudocolor plots.
- [x] A numerical value for number of cells or percentage (with statistics) is provided.

Methodology

Sample preparation	Mice where then euthanized via overdose of pentobarbital and blood was collected in EDTA coated syringes by cardiac puncture. RBC lysis of the blood was carried out and samples were centrifuged at 450g for 5 min at 4˚C. Bone marrow was collected by flushing a femur and spleen was homogenized.
Instrument	Acquisition was performed on BDFortessa using FacsDiva software (BD Bioscience)
Software	FlowJo
Cell population abundance	~30% of Ly6G-positive CD11b-positive cells in the blood are neutrophils; ~ 20% of Ly6G-positive CD11b-positive cells in the spleen are neutrophils: ~ 60% of Ly6G-positive CD11b-positive cells in the BM are neutrophils.
Gating strategy	Attached

- [x] Tick this box to confirm that a figure exemplifying the gating strategy is provided in the Supplementary Information.
